# Network pharmacology and in vivo experiments reveal the pharmacological effects and molecular mechanisms of Simiao Powder in prevention and treatment for gout

**DOI:** 10.1186/s12906-022-03622-0

**Published:** 2022-06-07

**Authors:** Huachong Xu, Jialin Wu, Shiqi Wang, Lu Xu, Pei Liu, Yucong Shi, Sizhi Wu, Li Deng, Xiaoyin Chen

**Affiliations:** College of Traditional Chinese Medicine, Jinan University, Guangzhou, 510632 China

**Keywords:** Simiao Powder, Gout, Traditional Chinese medicine, Network pharmacology

## Abstract

**Background:**

Gout is a common disease with high incidence due to unhealthy diet and living habits. Simiao Powder, as a classic formula consisted of four common herbs, has been widely used in clinical practice since ancient times to prevent and treat gout. However, the pharmacological mechanism of Simiao Powder is still unclear.

**Methods:**

Based on network pharmacology, Simiao Powder active compounds were identified in TCMSP, ETCM and BATMAN database, used to establish a network of interaction between potential targets of Simiao Powder and known therapeutic targets of gout. Subsequently, the key potential targets are being used for protein–protein interaction, GO enrichment analysis and KEGG pathway enrichment analysis through several authoritative open databases. Molecular docking through AutoDockTools software can verify interaction between molecules. Finally, to validate the predicted results, in vivo experiments based on hyperuricemic-gout mice model were designed and treated with Simiao powder and allopurinol. Serum levels of uric acid (UA), creatinine (Cr), blood urea nitrogen (BUN) and xanthine oxidase (XOD) were determined using a customized assay kit while the expression of PPAR-γ, PTGS1, IL-6 and Bcl2 mRNA were analyzed through qRT-PCR.

**Results:**

Disease-target-compound network was visualized basing on the 20 bioactive compounds and the 19 potential targets using Cytoscape software. The results of PPI analysis, GO enrichment and KEGG pathway enrichment analysis indicate that the potential mechanism of Simiao Powder in treating gout may be achieved by regulating immune and inflammatory reactions, improving metabolism and endocrine. The results of molecular docking show that most of the targets and components have good binding activity. In vivo experiments revealed that Simiao powder can decreased serum UA and XOD levels in hyperuricemic-gout mice, and improved renal function. Furthermore, Simiao powder certainly regulates the expression of PPAR-γ, PTGS1, IL-6 and Bcl2 mRNA in ankle tissue in hyperuricemic-gout mice.

**Conclusion:**

Collectively, this research predicted a multiple compounds, targets, and pathways model mechanism of Simiao Powder in the prevention and treatment of gout, providing new ideas and methods for in-depth research, via vivo experiments.

## Introduction

Gout is a common chronic disease with urate crystal deposition in and around the joints results from longstanding hyperuricaemia [1, 2]. The general incidence of gout is 1–4% of the general population [3] and the worldwide incidence of gout is reported to increase gradually due to poor dietary habits [4, 5], improved medical care and increased longevity [6]. Some studies [7, 8] with consistent methods of case ascertainment showed a rise more than 1% in the prevalence of gout in US and UK while the prevalence in 2008 was estimated at 1·14% in eastern China, where gout was regarded as a very rare disease in 1980 [5]. Because of the need for long-term treatment, gout not only poses a huge physical and financial burden on patients, but also increases the risk of co-occurring diseases, including cardiovascular [9, 10], metabolic and endocrine diseases [11, 12].

The treatment of gout mainly includes anti-inflammatory and analgesic treatment in acute arthritis, uric acid control in chronic phase and daily personal life management [13]. Medications for acute gout attacks mainly contain colchicine, Non-Steroidal anti-inflammatory Drugs (NSAIDs), steroids and biological agents [13]. Although the above drugs are recommended as first-line drugs for clinical treatment of gout, they are restricted to different degrees due to their respective liver and kidney toxicity and gastrointestinal adverse reactions [3]. Biological agents, such as interleukin-1 blockers, can achieve better therapeutic results with minimal side effects [3, 14], but are difficult to popularize because of their high cost. Treatment in the asymptomatic and hyperuricemia stages of gout aims to control uric acid by reducing uric acid synthesis (including allopurinol and febuxostat) and increasing renal urate excretion (including probenecid, benzbromarone and URAT1 inhibitor) [3]. Lowering uric acid requires long-term persistence, but most of the above drugs are difficult to use for a long time due to toxic and side effects. Therefore, more natural products need to be found to treat gout.

At present, it has become the focus of pharmaceutical research to search for drugs to treat gout and reduce hyperuricemia from Chinese herbs and natural materials [15–17]. Recent years, Simiao Powder, which mainly consist of *Atractylodes Lancea (Thunb.)Dc*, *Phellodendri Chinrnsis Cortex*, *Cyathulae Radix*, *Coicis Semen*, is derived from traditional Chinese medicine and widely used in the clinical treatment of gout in China due to good efficacy and few side effects [18, 19]. Modern pharmacology studies have also shown that Simiao Powder have obvious anti-inflammatory effects and inhibit the activity of xanthine oxidase for gout treatment [20–22]. However, the specific mechanism of Simiao Powder in treating gout has not been fully studied. In particular, the treatment of gout by Simiao Powder may be a multi-component-target-pathway model since the pathogenesis of gout is related to immune regulation, inflammatory response and metabolism.

**Fig. 1:**
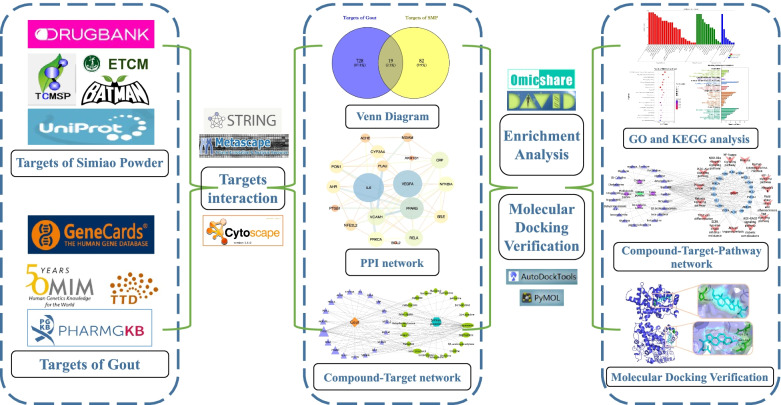
Workflow of the study design

Network pharmacology is a new method of drug research proposed based on multi-directional systematic pharmacology and biology [23–25], which combines drug component target network with biological target network from multiple perspectives to study the mechanism of drug action more systematically and comprehensively. Therefore, this study used network pharmacology method to explore the potential mechanism of Simiao Powder in the treatment of gout at multiple levels of compound-target-pathway. The experimental procedures of our study were shown in Fig. 1.

## Materials and methods

### Identification and screening of chemical compounds

To identify the chemical compounds of the four herbs in Simiao Powder, we performed a search in Traditional Chinese Medicine Systems Pharmacology Database and Analysis Platform [26] (TCMSP, http://lsp.nwu.edu.cn/tcmsp.php), ETCM [27] (http://www.tcmip.cn/ETCM/index.php/Home), BATMAN [28] (http://bionet.ncps-b.org.cn/batman-tcm) using “Atractylodes Lancea (Thunb.)Dc”, “Phellodendri Chinrnsis Cortex”, “Cyathulae Radix” and “Coicis Semen”. TCMSP, a unique systems pharmacology database for users to comprehensively study Chinese herbal medicines, contains information on all 500 TCM formulae registered in the Chinese Pharmacopoeia (2010 edition), with a total of 30,069 compounds extracted by literature mining and database integration [29].

According to ADME (absorption, distribution, metabolism and excretion) evaluation, two ADME-related models, including the evaluation of oral bioavailability (OB) and drug-likeness (DL), were employed to identify the potential bioactive compounds of Simiao Powder. Oral bioavailability (OB) and drug-likeness (DL) are very important pharmacokinetic parameters and key indicators in ADME process [29]. According to existing studies, OB ≥ 30% and DL ≥ 0.18 were used as screening criteria for drug compounds in this study.

### Identification of targets for chemical compounds

The protein targets of the compounds were searched from the TCMSP and Drugbank database [30] (https://www.drugbank.ca/) while the gene names were extracted from UniProt Knowledgebase [31] (UniProtKB, http://www.uniprot.org). The DrugBank database is a bioinformatics and cheminformatics resource that combines detailed drug data with comprehensive drug target information. The UniProtKB is a central hub for the collection of functional information on proteins, with accurate, consistent and rich annotation, includes widely accepted biological ontologies, classifications and cross-references, and clear indications of the quality of annotation in the form of evidence attribution of experimental and computational data.

### Identification of known therapeutic targets for gout

Therapeutic targets of gout were collected from four sources, include GeneCards database (https://www.genecards.org), the therapeutic target database [32] (TTD, https://db.idrblab.org/ttd/), Pharmacogenomics Knowledge Base [33] (PharmGKB, https://www.pharmgkb.org/), and the Online Mendelian Inheritance in Man (OMIM) database [34] (http:// www.omim.org/). The three databases mentioned above are commonly used databases for analysis of genomic known diseases with a genetic component and linked them to the relevant genes in the human genome, and they complement each other with their own characteristics. “Gout” was used to search in all the database as the keyword.

### Network construction of targets between Simiao Powder and gout

Firstly, the targets of active components of Simiao Powder were summarized, and the data were imported into the Cytoscape 3.6.0 software to construct the network of active component targets. Secondly, gout related gene targets obtained from the database were imported into Cytoscape 3.6.0 software to construct disease target network. Finally, the mapping function in Cytoscape 3.6.0 was used to combine the two networks to obtain the prediction targets that Simiao Powder might play a role in gout. This network can illustrate the relationships between putative targets of Simiao Powder and known therapeutic targets of gout.

### Protein–protein interaction

Based on the prediction targets between compounds and disease, protein–protein interaction (PPI) data were generated through two major existing public PPI databases with the species limited as “Homo sapiens”, including Metascape [35] (http://metascape.org/) and String [36] (https://string-db.org). The two open databases covered the majority of known protein–protein interactions information for human.

### GO enrichment analysis and pathway enrichment analysis

The gene ontology (GO) enrichment analysis was conducted to further validate whether the potential targets are indeed a match for gout in three levels, contained biological process analysis, molecular function analysis, cell component analysis. The GO enrichment analysis was performed using the functional annotation tool of DAVID Bioinformatics Resources 6.7 [37] (https://david-d.ncifcrf.gov/) and OmicShare platform (https://www.omicshare.com/).

To comprehensively understand the pathway mechanisms of Simiao Powder, the KEGG pathway enrichment analysis was also conducted using the functional annotation tool of DAVID Bioinformatics Resources 6.7 and OmicShare platform. Finally, Target-pathway network for Simiao Powder acting on gout was constructed by Cytoscape 3.6.0 ClueGO.

The pink and blue nodes represent the pathway and targets, respectively, and the edges represent the interactions among them.

### Molecular docking verification

The analysis obtained the targets with degree value ≥ 6 in the Disease-Target- Compound Network diagram and the key protein targets from PPI network. These targets were searched for literature, and finally screened out with gout-related targets, docking with the active compounds of Simiao Powder. The binding strength and activity between the target and the active compound were evaluated based on the binding energy and the hydrogen bond.

### Animals and preparation of Simiao Powder liquor

Male Kun-Ming mice (20 ± 2 g) was purchased from Liaoning Changsheng Biotechnology Co., Ltd. (Benxi, Liaoning Province, PR China). Animals were housed under a normal 12-h/12-h light/dark schedule, room temperature (22 ± 2 °C), relative humidity (55 ± 5%), and given a standard chow and water ad libitum for the duration of the study. All studies were carried out in accordance with the Institutional Animal Care Committee at the Jinan University. Simiao Powder, consisting of four classical herbs: Atractylodes Lancea (Thunb.)Dc, Phellodendri Chinrnsis Cortex, Cyathulae Radix, Coicis Semen,were purchased from Huarun Sanjiu Medicine Co., Ltd. According to the literature [38], it was recommended that the dosages of the four herbs granules in Simiao Powder were as follows: 15 g, 15 g, 15 g and 30 g. According to the different compatibility ratios of Simiao Powder, the four herbs were mixed, weighing 75 g in total.

### Hyperuricemic-gout mice and drug administration

Experimental animal model of Hyperuricemic-Gout Mice induced by uricase inhibitor potassium oxonate has been used to study drug action for many years. Mice were divided into 4 groups: Normal + water(group A), Model + water(group B), Model + Simiao Powder(group C), Model + allopurinol(group D). The normal group was intraperitoneally injected with 0.5% CMC-Na solution, while the other groups were intraperitoneally injected with suspension composed of 300 mg/kg potassium oxazinate and 200 mg/kg hypoxanthine at 15:00 p.m. every day for seven consecutive days (reagents purchased from Sigma, St. Louis, MO, USA). Simiao Powder and allopurinol were orally initiated at 16:00 p.m. on the day by intragastric administration. Dosages of Simiao powder and allopurinol were determined based on the conversions from clinical adult dosages and previous studies. The dosage was as follows [38]: allopurinol 20 mg/kg/day, Simiao Powder 11.3 g/kg/day. The clinical efficacy of Simiao powder was generally evaluated after a week of treatment, so we chose 7 days as the treatment time.

### Blood and ankle tissue sample collection

Whole blood samples were collected 1 h after final administration on the seventh day, and then allowed to clot for approximately 1 h at room temperature and centrifuged at 3500 rpm for 10 min to obtain serum, stored at − 80 °C until assayed. Simultaneously, ankle tissue was rapidly and carefully separated on ice-plate and stored at − 80 °C for assays, respectively.

### Determination of serum uric acid, creatinine, blood urea nitrogen and xanthine oxidase levels

Serum levels of uric acid (UA), creatinine (Cr), blood urea nitrogen (BUN) and xanthine oxidase (XOD) were determined using a customized assay kit. All assay kits were purchased from Jiancheng Biotech. (Nanjing, PR China). Uric acid concentration in serum was determined by the phosphotungstic acid method. Blood urea nitrogen was determined by rapid urease method while Creatinine was measured using sarcosine oxidase method. Xanthine Oxidase was detected by colorimetric method. The operation method of the experiment shall strictly refer to the manufacturer's instructions.

### RT-PCR of PPAR-γ, PTGS1, IL-6, and Bcl2

Related methods are described in our previous article [39]. Briefly, Total RNA was extracted by RNAiso Plus (catalog No.9108; TaKaRa, Kusatsu, Japan) according to the manufacturer’s instructions. The cDNA synthesis and RT-qPCRs were conducted using a CFX Connect Real-Time PCR Detection system (Bio-Rad, Berkeley, CA, USA) with PrimeScript RT reagent kits (RR047A; TaKaRa) and SYBR Premix EX Taq II (RR820A; TaKaRa) according to the manufacturer’s instructions. The primers were synthesized by Generay Biotech (Shanghai, China), and the primers for RT-qPCR are listed in Table 1. The transcript levels were normalized to an internal control (GAPDH).

**Table 1:** The primers for RT-qPCR

Gene name	Primers ( 5’-3’)
IL-6	Forward: CTGCAAGAGACTTCCATCCAG Reverse: AGTGGTATAGACAGGTCTGTTGG
PPAR-γ	Forward: GGAAGACCACTCGCATTCCTT Reverse: GTAATCAGCAACCATTGGGTCA
PTGS1	Forward: ATGAGTCGAAGGAGTCTCTCG Reverse: GCACGGATAGTAACAACAGGGA
Bcl2	Forward: GCTACCGTCGTGACTTCGC Reverse: CCCCACCGAACTCAAAGAAGG
GAPDH	Forward: TGATGACATCAAGAAGGTGGTGAAG Reverse: TCCTTGGAGGCCATGTAGGCCAT

### Statistical analysis

All data are statistically analyzed using one-way ANOVA analysis with the statistical software SPSS 13.0. Data were expressed as Mean ± Standard Error of the mean(S.E.M.). A value of *P* < 0.05 was considered statistically significant. Figures were obtained by GraphPad Prism 9 (GraphPad Software, Inc., San Diego, CA).

## Results

### Active chemical compounds of Simiao Powder

Based on the criteria of OB ≥ 30% and DL ≥ 0.18, a total of 59 compounds of Simiao Powder were retrieved from the TCMSP, including 9 in *Atractylodes Lancea (Thunb.)Dc*, 4 in *Phellodendri Chinrnsis Cortex*, 37 in *Cyathulae Radix*, 9 in *Coicis Semen*. Among the 59 satisfied compounds, a total of 56 compounds were finally obtained after excluding the duplicates.

### Putative targets for Simiao Powder

According to the target prediction system in TCMSP and Drugbank databases, the 56 potential active components of Simiao Powder collected were input into the databases and 824 putative targets were obtained by pairing. The corresponding target proteins were searched through the UniprotKB database for their corresponding gene symbol names.

### Known therapeutic targets for gout and disease-drug common targets

A total of 756 targets associated with gout were collected from the four open databases, namely, 734 in GeneCards database, 15 in TTD, 4 in PharmGKB and 3 in OMIM database. After removing the overlapping targets, 746 associated targets were obtained.

**Fig. 2:**
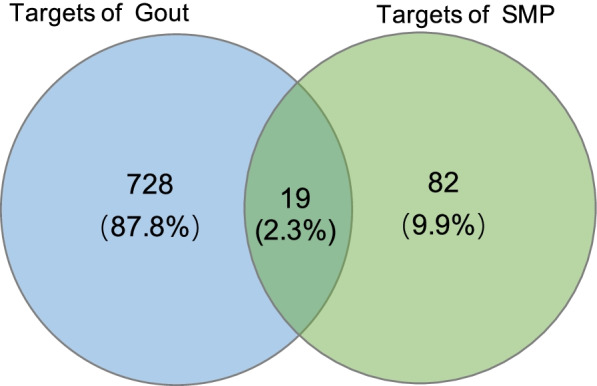
Intersection targets of Simiao Powder and gout

The prediction targets of compounds and disease-related targets were combined and intersected to obtain the possible targets of drug treatment for gout. Subsequently, the result showed that gout share 19 targets with 20 bioactive compounds of Simiao Powder (Fig. 2). The 19 targets are the common targets between gout and 20 bioactive compounds. The detailed information of the associated targets and 20 bioactive compounds are provided in Tables 2 and 3, respectively.

**Table 2:** Information regarding gout-related targets of Simiao Powder

Number	Target ID	Target name	Gene
1	TAR00006	Prostaglandin G/H synthase 1	PTGS1
2	TAR00078	Peroxisome proliferator activated receptor gamma	PPARG
3	TAR04565	Transcription factor p65	RELA
4	TAR00086	Apoptosis regulator Bcl-2	BCL2
5	TAR00351	Interleukin-6	IL6
6	TAR00165	Acetylcholinesterase	ACHE
7	TAR04469	Protein kinase C alpha type	PRKCA
8	TAR00357	Serum paraoxonase/arylesterase 1	PON1
9	TAR00288	Aldose reductase	AKR1B1
10	TAR00740	Vascular endothelial growth factor A	VEGFA
11	TAR00346	Urokinase-type plasminogen activator	PLAU
12	TAR04394	NF-kappa-B inhibitor alpha	NFKBIA
13	TAR00621	Cytochrome P450 3A4	CYP3A4
14	TAR00427	E-selectin	SELE
15	TAR00440	Vascular cell adhesion protein 1	VCAM1
16	TAR00318	Maltase-glucoamylase, intestinal	MGAM
17	TAR04398	Nuclear factor erythroid 2-related factor 2	NFE2L2
18	TAR03204	Aryl hydrocarbon receptor	AHR
19	TAR04130	C-reactive protein	CRP

**Table 3:** Bioactive Compounds of Simiao Powder related to Gout

MOL ID	MOL Name	OB (%)	DL
MOL000173	wogonin	30.68	0.23
MOL000188	3β-acetoxyatractylone	40.57	0.22
MOL012286	Betavulgarin	68.75	0.39
MOL000359	beta-sitosterol	36.91	0.75
MOL000098	quercetin	46.43	0.28
MOL001454	berberine	36.86	0.78
MOL001458	coptisine	30.67	0.86
MOL002651	Dehydrotanshinone II A	43.76	0.40
MOL002662	rutaecarpine	40.30	0.60
MOL002666	Chelerythrine	34.18	0.78
MOL000449	Stigmasterol	43.83	0.76
MOL002668	Worenine	45.83	0.87
MOL002670	Cavidine	35.64	0.81
MOL000785	palmatine	64.60	0.65
MOL000787	Fumarine	59.26	0.83
MOL000790	Isocorypalmine	35.77	0.59
MOL001455	(S)-Canadine	53.83	0.77
MOL002894	berberrubine	35.74	0.73
MOL006422	thalifendine	44.41	0.73
MOL001494	Mandenol	42.00	0.19

### Disease-target- compound network and analysis

**Fig. 3:**
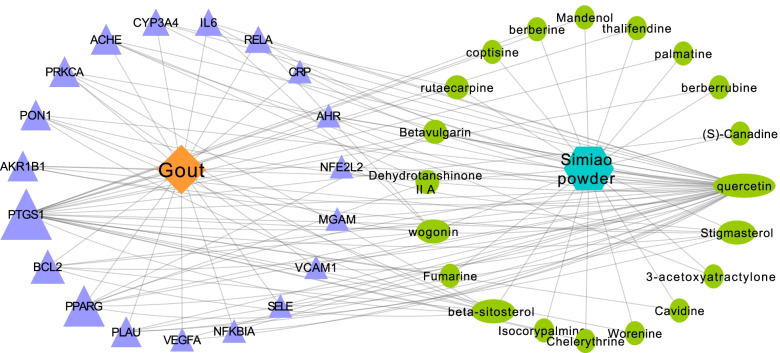
Disease-Target-Compound Network of Simiao Powder and Gout. The orange, purple, green and cyan nodes represent the disease, targets, compounds and formula, respectively; the edges represent the interactions among them and nodes sizes are proportional to their degree

Disease-Target- Compound network was visualized on the basis of the 20 bioactive compounds and the 19 combined targets using Cytoscape software. As shown in Fig. 3, the network is consisted of 41 nodes (1 disease, 1 formula, 19 targets and 20 bioactive compounds). The orange, purple, green and cyan nodes represent the disease, targets, compounds and formula, respectively; the edges represent the interactions among them and nodes sizes are proportional to their degree. Furthermore, network analysis was performed to evaluate the centralization and heterogeneity [29]. The resulting centralization and heterogeneity of the network are 0.412 and 1.170, respectively, indicating that some nodes are more concentrated in the network than others. In brief, the compound-target space is biased toward certain compounds and targets [29], which means that some compounds have multiple targets or the target has multiple compounds, especially the high-degree compounds such as MOL000098 (quercetin, degree = 41), MOL000359 (beta-sitosterol, degree = 9), MOL000449 (stigmasterol, degree = 7), MOL000173 (wogonin, degree = 6) and the high-degree targets such as PTGS1(degree = 22), PPARG(degree = 8), BCL2(degree = 6).

### Protein–protein interaction analysis

**Fig. 4:**
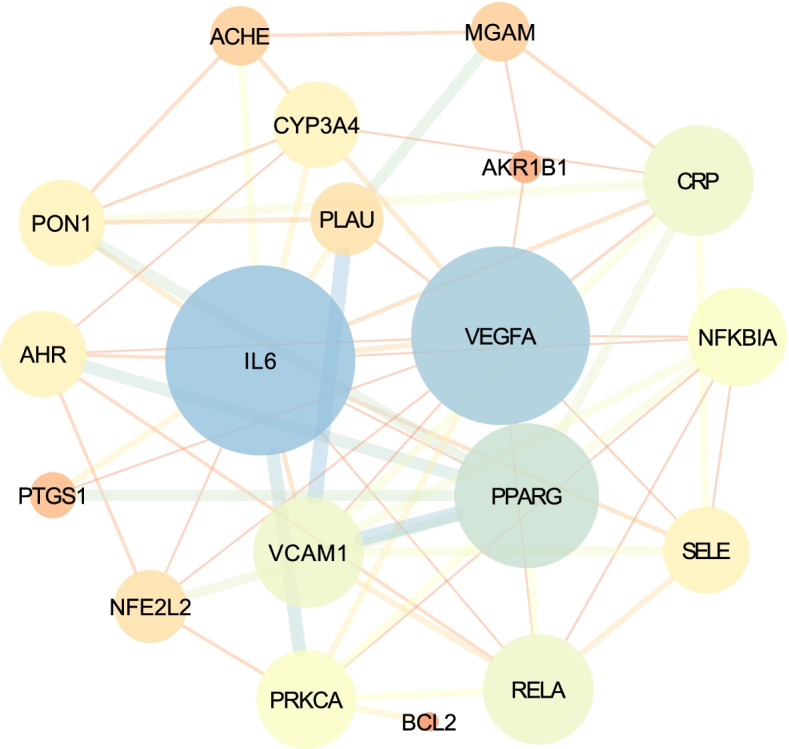
The PPI network of gout-related targets of Simiao Powder. Note: the circular nodes represent the interacting proteins that directly or indirectly interact with each other. The node size represents the importance of proteins, and the thickness of the line represents the closeness of the relationship between proteins

Based on the prediction targets between compounds and disease, protein–protein interaction (PPI) data were generated for the 19 common targets mentioned above (The confidence score > 0.4). The PPI network showed current interactions among the 19 common targets (Fig. 4). Network nodes represent proteins, splice isoforms or post-translational modifications are collapsed, ie. each node represents all the proteins produced by a single, protein-coding gene locus [36]. Edges represent protein–protein associations which are meant to be specific and meaningful and line thickness indicates the strength of data support. As is shown in Fig. 4, the key protein targets include IL-6, PTGS1, PPARG, NFKBIA, AHR, PRKCA and VEGFA.

### GO enrichment analysis for targets

**Fig. 5:**
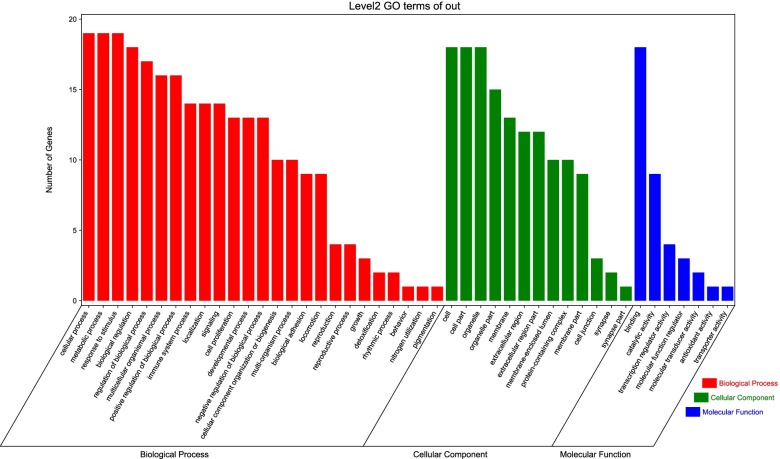
The annotation clustering result of GO enrichment analysis

**Fig. 6:**
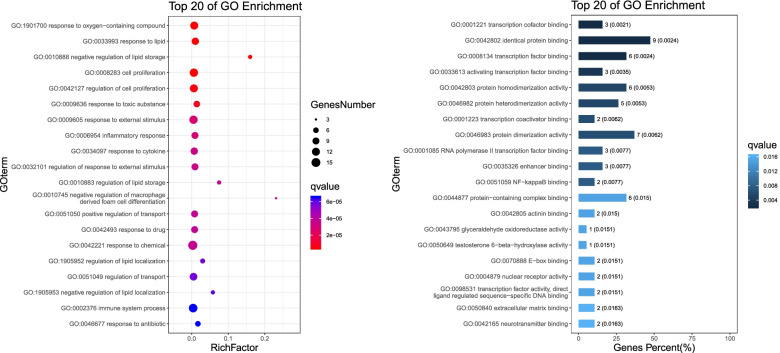
Top 20 of GO enrichment terms of biological process and molecular function

The gene ontology (GO) enrichment analysis was performed and the results were revealed in three levels via biological process, molecular function and cell component analysis. Figure 5 shows the annotation clustering result of GO enrichment analysis. At the level of molecular function, Simiao Powder has a great influence on protein binding, catalytic activity and transcriptional regulatory activity; at the level of cell components, Simiao Powder has a great influence on cells, mainly including cell parts, cytoplasm, organelles and cell membranes; In terms of biological processes, Simiao Powder mainly affects cellular process, metabolic process, response to stimulus, biological regulation process, immune system process, and multicellular organismal process etc. Figure 6 list the 20 most significantly enriched GO terms of biological process and molecular function respectively. Notably, numerous targets are involved in cellular process, metabolic process, response to stimulus, biological regulation process and immune system process, which are closely related to the pathogenesis of gout.

### The KEGG pathway enrichment analysis

**Fig. 7:**
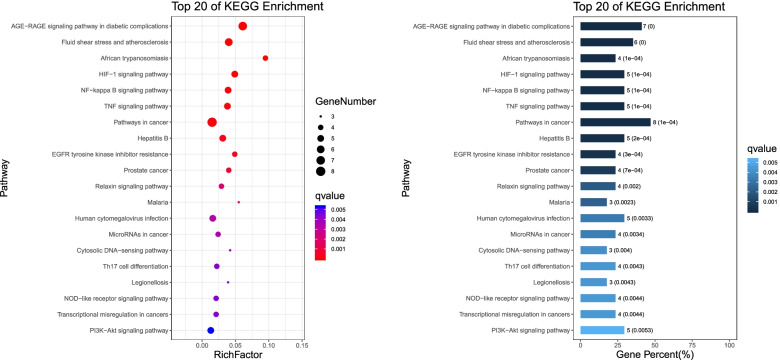
Top 20 of KEGG pathways enrichment with Rich-Factor and Gene Percent

**Fig. 8:**
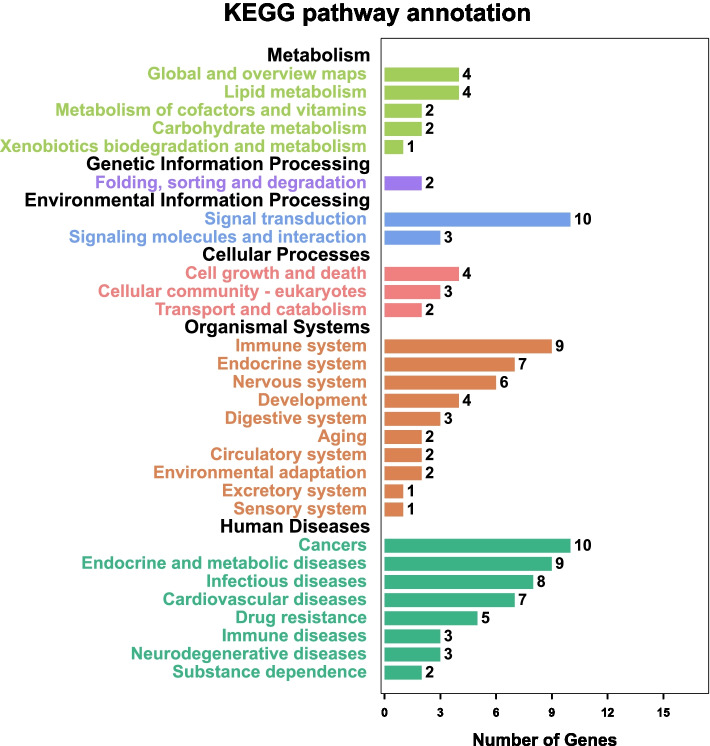
KEGG pathway annotation of gout-related targets of Simiao Powder

The KEGG pathway enrichment analysis was performed using the functional annotation tool of DAVID Bioinformatics Resources 6.7 and OmicShare platform. Figure 7 list the 20 most significantly enriched KEGG pathways with RichFactor and Gene Percent respectively. It shows that Simiao Powder achieves its therapeutic effect for gout treatment through multiple pathways, mainly including AGE-RAGE signaling pathway in diabetic complications, Fluid shear stress and atherosclerosis, HIF-1 signaling pathway, NF-kappa B signaling pathway, TNF signaling pathway, EGFR tyrosine kinase inhibitor resistance, Cytosolic DNA-sensing pathway, Th17 cell differentiation Legionellosis, NOD-like receptor signaling pathway、PI3K-Akt signaling pathway. To further elucidate the mechanisms of Simiao Powder for gout treatment, we constructed a functional annotation clustering for significant KEGG pathways as shown in Fig. 8. Moreover, the results reveal that related pathways are mainly concentrated in the aspects of immune and endocrine systems, lipid metabolism, cofactor and vitamin metabolism, signal transduction and cardiovascular diseases, which are considered closely associated with gout.

**Fig. 9:**
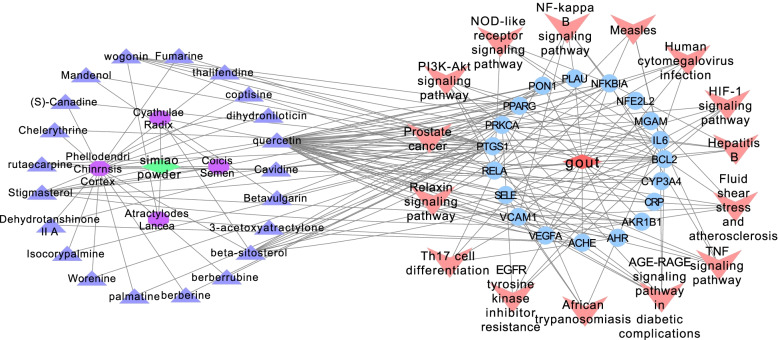
Compounds-Targets-pathways network for Simiao Powder acting on gout

Finally, Compounds-Targets-pathways network for Simiao Powder acting on gout was constructed shown as Fig. 9.

### Molecular docking verification

The molecular docking software AutoDockTools can apply semi-flexible docking methods on the basis of complex networks to study the interaction between molecules, and then evaluate the protein–ligand binding potential based on its comprehensive characteristics. There were 3 targets with Degree value ≥ 6 in the disease-target-compound network diagram, and top 3 targets in the PPI network diagram. After removing duplicate targets, we obtained 4 targets in total: IL-6, PTGS1, PPARG, BCL2. These targets were searched in the literature respectively and all targets were related to gout. They were respectively molecularly docked with the potential active compounds of Simiao Powder.

**Fig. 10:**
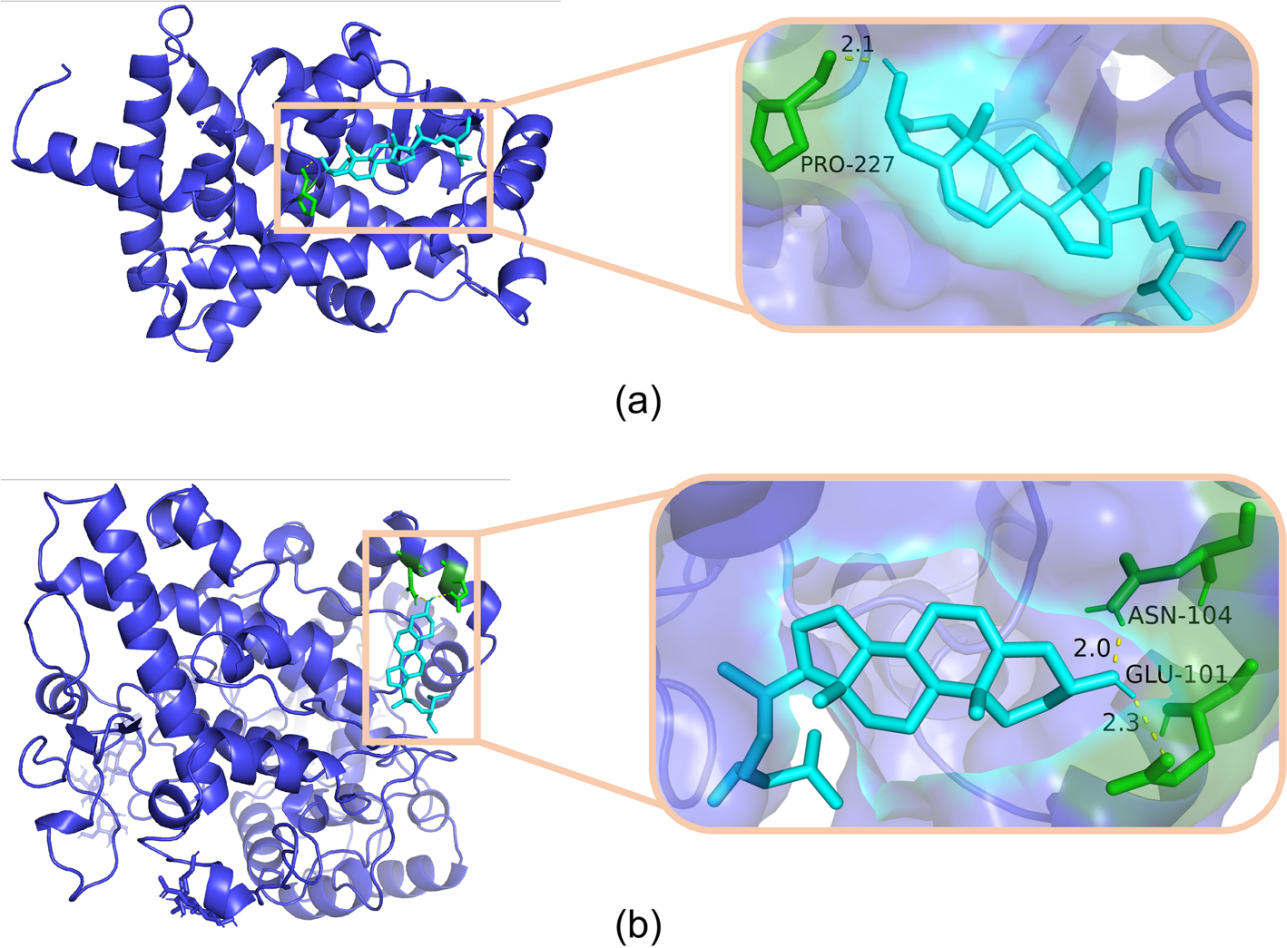
Docking results for molecules with binding energy lower than -7 kcal/mol. Note: (**a**) the molecular docking result of PPARG and stigmasterol, the number of hydrogen bonds: 1, the binding energy: -7.72 kcal/mol; (**b**) the molecular docking result of PTGS1 and beta-sitosterol, the number of hydrogen bonds: 1, the binding energy: -7.01 kcal/mol

According to the results of comprehensive molecular docking, the minimum binding energy was -3.31 kcal/mol and the maximum is -7.72 kcal/mol. Among them, the 4 potential active compounds of Simiao Powder (quercetin, beta-sitosterol, stigmasterol, wogonin) and the amino acid residues of the targets IL-6, PTGS1, PPARG, BCL2 were combined by intermolecular hydrogen bonds. Most of the targets and components had good binding activity because of high binding energy. This indicates that the main characteristic chemical components of Simiao Powder have good binding ability with the targets IL-6, PTGS1, PPARG and BCL2. In the results of molecular docking, there were 31.25% of them binding energy higher than -5 kcal/mol while others were lower than -5 kcal/mol. In addition, there were two of them lower than -7 kcal/mol. Among them, the binding energy of PPARG and stigmasterol is the highest, -7.72 kcal/mol. The results of molecular docking were shown in Table 4. As shown in Fig. 10, the docking results with binding energy lower than -7 kcal/mol were below, which hydrogen bonds and the binding site were drawn by PyMOL.

**Table 4:** The results of molecular docking

Targets	Compounds	Binding energy
IL-6	quercetin	-4.20 kcal/mol
IL-6	beta-sitosterol	-5.48 kcal/mol
IL-6	stigmasterol	-6.33 kcal/mol
IL-6	wogonin	-5.71 kcal/mol
PPARG	quercetin	-3.72 kcal/mol
PPARG	beta-sitosterol	-6.31 kcal/mol
PPARG	stigmasterol	-7.72 kcal/mol
PPARG	wogonin	-5.74 kcal/mol
PTGS1	quercetin	-4.72 kcal/mol
PTGS1	beta-sitosterol	-7.01 kcal/mol
PTGS1	stigmasterol	-6.71 kcal/mol
PTGS1	wogonin	-6.40 kcal/mol
BCL2	quercetin	-3.31 kcal/mol
BCL2	beta-sitosterol	-6.02 kcal/mol
BCL2	stigmasterol	-6.15 kcal/mol
BCL2	wogonin	-4.86 kcal/mol

### Simiao powder decreased serum UA and XOD levels in hyperuricemic-gout mice

**Fig. 11:**
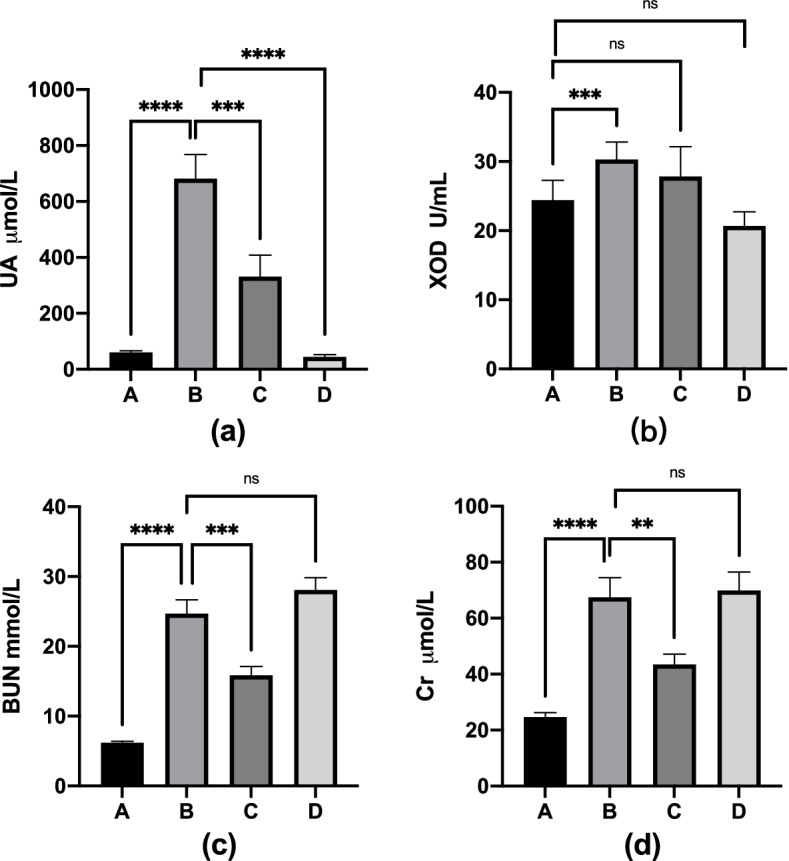
The effects of Simiao powder and allopurinol on serum levels of UA, XOD, BUN and Cr. (*P < 0.05, **P < 0.01, ***P < 0.001, ****P < 0.0001.)

As expected, intraperitoneally injected potassium oxazinate and hypoxanthine significantly elevated serum UA and XOD levels compared with normal mice (Fig. 11a). Simiao powder significantly decreased serum UA levels of hyperuricemic-gout mice to the normal while allopurinol, as a positive control, reduced the levels of hyperuricemic mice, even lower than that of normal group (Fig. 11a). Xanthine oxidase can catalyze hypoxanthine to xanthine and increase uric acid, and also directly catalyze xanthine to uric acid, which has great influence on serum uric acid production. Both allopurinol and Simiao powder can reduce the level of XOD in serum, but only allopurinol has a significant difference, even lower than the normal group (Fig. 11b).

### Simiao powder improved renal function in hyperuricemic-gout mice

Serum BUN and Cr levels in Hyperuricemic-gout mice were significantly higher than those in the normal group (Fig. 11c-d). Simiao powder can effectively reverse the changes of the above indexes and reduce serum Cr and BUN in hyperuricemia mice to the level close to the normal group (Fig. 11c-d). conversely, allopurinol cannot decrease serum Cr and BUN levels in disease models, even higher than that of model group (Fig. 11c-d).

### Simiao powder regulates the expression of PPAR-γ, PTGS1, IL-6 and Bcl2 mRNA in Ankle tissue in hyperuricemic-gout mice

**Fig. 12:**
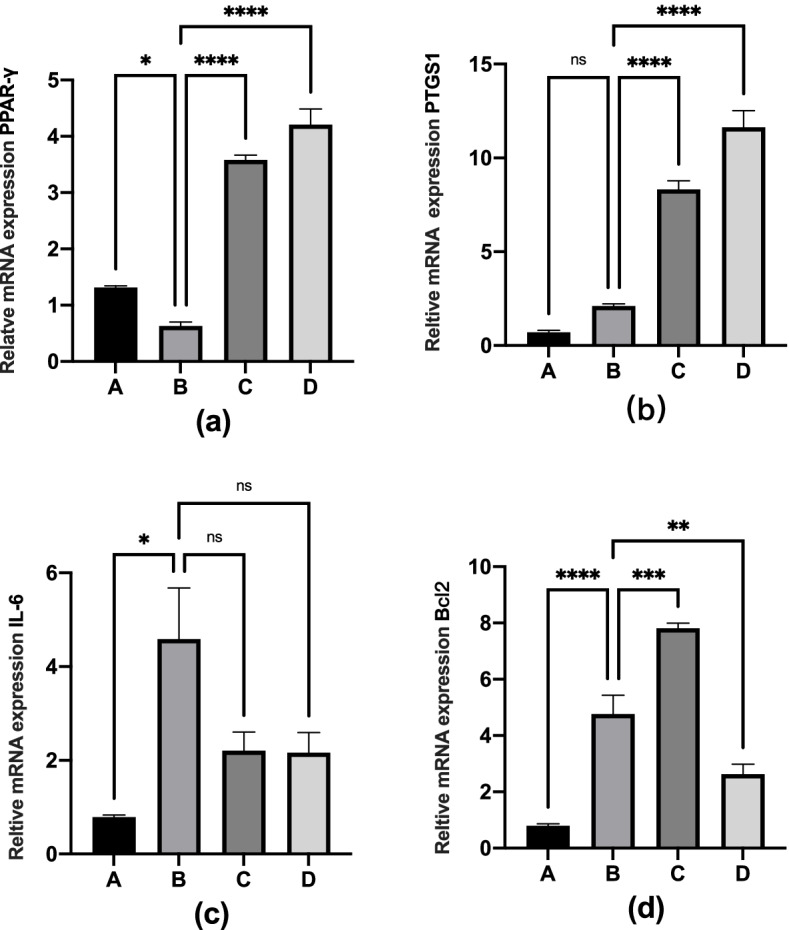
The effects of Simiao powder and allopurinol on ankle mRNA levels of PPAR-γ, PTGS1, IL-6 and Bcl2. (*P < 0.05, **P < 0.01, ***P < 0.001, ****P < 0.0001.)

The effects of Simiao powder and allopurinol on mRNA levels of ankle PPAR-γ, PTGS1, IL-6, and Bcl2 in hyperuricemic-gout and normal mice were shown in Fig. 12a-d. Compared with normal mice, the mRNA expression of PPAR-γ was significantly decreased in hyperuricemic-gout mice, but significantly increased after treatment with Chinese medicine or Western medicine, even higher than the normal group. PTGS1 mRNA were increased in hyperuricemic-gout mice compared with normal mice, but there was no significant difference. Both Simiao powder and allopurinol did not reduce the mRNA expression of PTGS1, even higher than model group. Compared with normal mice, the mRNA expression levels of IL-6 and Bcl2 were significantly increased in hyperuricemic-gout mice. Both Simiao powder and allopurinol can down-regulate the expression of IL-6 mRNA, but still not restore to the normal value. In addition, allopurinol can reduce the mRNA expression level of Bcl2 in oxonate-induced hyperuricemic mice, but Simiao powder bring an opposite effect.

## Discussion

Traditional Chinese medicine considers gout to be arthralgia syndrome result from the dysfunction of viscera [40], which is caused by the changes of poor dietary habits. There are similarities between Chinese and western medicine in understanding the causes of gout. Moreover, Gout is generally regarded as dampness and heat stagnation in meridians and muscles-bones [40], while Simiao Powder has the effect of clearing heat and dampness, relaxing muscles and clearing collaterals [19], and is a classic prescription for treating dampness and heat.

**Fig. 13:**
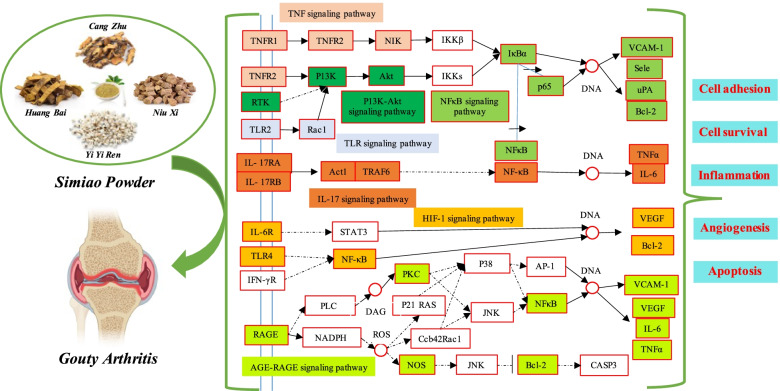
Potential pathways and therapeutic modules between Gout and Simiao Powder. Seven pathways (marked in different colors) constitute the compressed pathway

This research demonstrates that 20 active compounds and 19 potential genetic targets of Simiao Powder in the treatment of gout were found through network pharmacology. Then, specific functions and mechanisms are revealed through protein interaction network analysis, GO enrichment analysis and KEGG pathways enrichment analysis. The results showed that Simiao Powder, through multi-components-targets-pathways mode, play an important role on the immune regulation, inflammatory response, metabolism and endocrine regulation, cell signal transduction, cell growth and apoptosis, so as to achieve anti-inflammatory and analgesia, uric acid lowering and metabolism improvement. Further classification and analysis showed that the therapeutic effects of Simiao Powder on gout mainly focused on several modules, such as immune regulation and inflammatory response, metabolism and endocrine regulation, and cell function. To visually reflect the important pathway diagram and its key targets, they were compressed and drawn in Fig. 13, based on the signal pathway maps of the Kyoto Encyclopedia of Genes and Genomes database [41].

### Immune regulation and inflammatory response module

Generally speaking, uric acid crystal deposition in articular cavity is the trigger of gout [3, 13]. These crystals initiate the inflammatory process by being engulfed by synovial phagocytes, leading to the release of lysosomal enzymes and the production of inflammatory chemokines [3]. Another mechanism is that uric acid crystal changes the stability of phagocytes membrane by directly crosslinking with membrane lipids and glycoproteins. The presence of cytokines, to as IL-1β, IL-6 and TNF-a, suggest that these cytokines have a role in granuloma formation in to patients [42]. Colchicine is thought to prevent acute attacks by altering the affinity of selectin on endothelial cells and neutrophils to inflammatory mediators and by blocking eutrophic stimulation induced by endothelial cells [43].

Among the major putative targets and pathways of Simiao Powder, most of them are related to the immune regulation and inflammatory response for gout. At the gene target level, PTGS1 is the key target with the largest number of junctions, usually responds to hormone stimulation rapidly to produce prostaglandins and continuously regulate physiological processes, such as mediating responses to inflammatory stress [44]. Furthermore, deletion of PTGS1 substantially repressed the expressions of NF-κB genes such as IL6, IL8, and SELE [45]. RELA can encodes a nuclear transcription factor-κB (NF-κB) p65 subunit protein and participate in the formation of NF-κB dimers [46]. NF-κB capture in the cytoplasm REL dimers, thus inhibiting dimers NF-kappa B/REL the activity of the complexes. Almost all types cell is affected by transcription factors, which is considered as the end of a series of signal transduction events. Many biological processes such as inflammation, immunity, cell growth and apoptosis are closely related to NF-κB. Another target IL-6 is an important mediator of febrile and acute responses [47]. At the pathway level, five key signaling pathways, namely, NF-kappa B signaling pathway, TNF signaling pathway, Th17 cell differentiation legionel disease, Nod-like receptor signaling pathway and Cytosolic DNA-sensing pathway, were all closely associated with immune and inflammatory reactions.

### Metabolism and endocrine regulation module

Gout is defined as a metabolic disorder [2]. Studies suggest that metabolic and endocrine regulation may play a role in gout attacks, and that obesity led to an increase in uric acid and exacerbate the risk of hyperuricemia [48–50]. In this study, PTGS1 and PPARG were the major targets related to metabolism and endocrine. PTGS1 is involved in the prostaglandin biosynthesis pathway as part of lipid metabolism [51, 52]. PPARG is a key regulator of fat cell differentiation and glucose homeostasis by regulating the peroxidosome beta oxidation pathway of fatty acids [53, 54]. It can also regulate the homeostasis of the intestinal environment through inflammation mediated by NF-κB [55]. Pathway enrichment results show that metabolic and endocrine pathways are important in the treatment. Especially, AGE-RAGE signaling pathway in diabetic complications is closely related to blood glucose and blood lipids [56]. Moreover, this pathway can further activate NF-κB activity by activating various intracellular signaling pathways of NADPH oxidase, protein kinase C and MAPK [57]. In addition, Vitamin C can increase the renal excretion of uric acid as a supplement during management of gout [58]. The enrichment of cofactor and vitamin metabolism also confirmed this idea.

### Cell function module

One mechanism of acute gout inflammation is the noninflammatory phagocytosis of macrophages to dying neutrophils [3, 59]. The pathogenesis involves initial activation of monocytes and mast cells, followed by neutrophils [3, 60, 61]. Well-differentiated macrophages have the ability to swallow urate crystals without provoking an inflammatory response. However, monocytes with low differentiation degree produce a large amount of TNF, IL-1, IL-6 and IL-8 after phagocytosis of urate crystals, and activate endothelial cells [3]. Activation of endothelial cells further exacerbates the inflammatory response and migration of neutrophils. Notably, the key targets of this research mainly related to cell growth, apoptosis and signal transduction. For example, PTGS1 plays an important role in cell protection [62, 63] while AHR participate in the regulation of cell cycle [64]. PRKCA is involved in cell proliferation, apoptosis, differentiation, migration and adhesion [65] while VEGFA can induce endothelial cell proliferation, promote cell migration, inhibit apoptosis and induce vascular through [66]. In terms of signaling pathways, HIF-1 signaling pathway and Cytosolic DNA-sensing pathway play an important role in cell signal transduction, while EGFR tyrosine kinase inhibitor resistance can regulate cell environment homeostasis [67].

### Multi-components-targets-pathways treatment model, molecular docking verification and research limitations

These modules have their own unique functions, but they are connected and affect with each other, forming a dynamic adjustment network. That reflects the characteristics of multiple compounds, multiple targets and multiple pathways of Simiao Powder in the treatment of gout. Furthermore, gout is often accompanied by other comorbidities [10, 11, 68, 69] and its treatment needs to be systematic and comprehensive. Many targets and pathways predicted in this study are related to cardiovascular system and metabolic system, which means Simiao Powder can give full consideration to the treatment of complications. In addition, the western medicine treatment of gout usually corresponds to the medication in stages and carefully controls the dosage [3], while Simiao Powder can be used for the treatment of gout in various stages due to its complex functional network.

Molecular docking reveals the interaction between components and targets in the network, thereby improving the accuracy of the network [70]. The negative score of the chemical bond binding energy is used to explain the docking result. The larger the absolute value of the negative score, the more stable the structure. According to the results of molecular docking, the binding energy of PPARG and stigmasterol is the lowest, -7.72 kcal/mol. According to above analysis, PPARG can regulate the homeostasis of the intestinal environment through inflammation, and stigmasterol can exert anti-inflammatory [71] and anti-cancer [72] effects. The binding energy of PTGS1 and beta-sitosterol is -7.01 kcal/mol. PTGS1 can also mediate responses to inflammatory stress. Researches have shown that beta-sitosterol has anti-inflammatory [73], antioxidant [74], anti-cancer [75] functions. According to the results of molecular docking, the ingredients of Simiao Powder can treat gout by exerting anti-inflammatory effects. In addition, the main characteristic chemical components of Simiao Powder have good binding ability with IL-6, PTGS1, PPARG and BCL2 targets, which indicates that the active components of Simiao Powder can effectively treat gout by combining with core targets.

### In vivo experiments demonstrated the efficacy of Simiao powder in the treatment of hyperuricemic-gout mice and identified key targets

Simiao powder decreased serum UA and XOD levels in hyperuricemic-gout mice, and improved renal function via regulating serum BUN and Cr levels. It was noted that both Simiao powder and allopurinol can effectively reduce serum uric acid and XOD, but Simiao powder may bring few side effects and improve renal function in the treatment. Our experimental results are consistent with a previous study [38]. In addition, our experiments showed that Simiao powder certainly regulates the expression of PPAR-γ, PTGS1, IL-6 and Bcl2 mRNA in ankle tissue in hyperuricemic-gout mice. These gene targets mentioned above are likely to affect the occurrence and development of gout and hyperuricemia through immune inflammation, apoptosis and endocrine system. However, an in-depth analysis of these targets mentioned above is still needed to elucidate their potential molecular mechanisms in Simiao powder treating gout.

Our research still has some limitations. For example, the actual absorption of drugs may be different from the reality, and drug compounds may react with each other to form new unknown compounds. Furthermore, our screening criteria for drug compounds are based on oral absorption and pharmacodynamics, but some compounds may not be absorbed into the blood but act on intestinal microecology, which is conducive to the regulation of immunity and metabolism.

## Conclusion

Collectively, this research predicted a multiple compounds, targets, and pathways model mechanism of Simiao Powder in the prevention and treatment of gout, providing new ideas and methods for in-depth research, via vivo experiments.

## Data Availability

The data used to support the findings of this study are available from the corresponding author upon request.

## Abbreviations

TCMSP: Traditional Chinese Medicine Systems Pharmacology
BATMAN-TCM: Tool for Molecular mechanism of Traditional Chinese Medicine
DAVID: Database for annotation, visualization and integrated discovery
OMIM: Online Mendelian Inheritance in Man
PPI: Protein–protein interaction
OB: Oral bioavailability
DL: Drug-likeness
GO: Gene Ontology
KEGG: Kyoto Encyclopedia of Genes and Genome
IL-6: Interleukin-6
PTGS1: Prostaglandin-endoperoxide synthase 1
PPARG: Peroxisome proliferator-activated receptor gamma
Bcl2: B-cell lymphoma-2
